# Metformin Treatment Prevents Sedentariness Related Damages in Mice

**DOI:** 10.1155/2016/8274689

**Published:** 2015-11-30

**Authors:** Pamela Senesi, Anna Montesano, Livio Luzi, Roberto Codella, Stefano Benedini, Ileana Terruzzi

**Affiliations:** ^1^Department of Biomedical Sciences for Health, University of Milan, Milan, Italy; ^2^Metabolism Research Center, San Donato Hospital and Scientific Institute, Milan, Italy; ^3^Diabetes Research Institute, Metabolism, Nutrigenomics and Cellular Differentiation Unit, San Raffaele Scientific Institute, Milan, Italy

## Abstract

Metformin (METF), historical antihyperglycemic drug, is a likely candidate for lifespan extension, treatment and prevention of sedentariness damages, insulin resistance, and obesity. Skeletal muscle is a highly adaptable tissue, capable of hypertrophy response to resistance training and of regeneration after damage. Aims of this work were to investigate METF ability to prevent sedentariness damage and to enhance skeletal muscle function. Sedentary 12-week-old C57BL/6 mice were treated with METF (250 mg/kg per day, in drinking water) for 60 days. METF role on skeletal muscle differentiation was studied *in vitro* using murine C2C12 myoblasts. Muscular performance evaluation revealed that METF enhanced mice physical performance (Estimated VO_2max_). Biochemical analyses of hepatic and muscular tissues indicated that in liver METF increased AMPK and CAMKII signaling. In contrast, METF inactivated ERKs, the principal kinases involved in hepatic stress. In skeletal muscle, METF activated AKT, key kinase in skeletal muscle mass maintenance. In *in vitro* studies, METF did not modify the C2C12 proliferation capacity, while it positively influenced the differentiation process and myotube maturation. In conclusion, our novel results suggest that METF has a positive action not only on the promotion of healthy aging but also on the prevention of sedentariness damages.

## 1. Introduction 

Type 2 diabetes mellitus is a metabolic disorder characterized by chronic hyperglycemia in association with insulin resistance, impaired relative and/or absolute insulin production, and altered glucagon secretion [1, 2]. At the onset of diabetes two main processes are involved in its pathogenesis: progressive decline in pancreatic islets function and reduced insulin sensitivity in peripheral tissues [3]. In particular, insulin resistance (IR) occurs when insulin effect on muscle and fat tissues glucose uptake is defective and is not capable of inhibiting endogenous glucose production by the liver [4]. Because skeletal muscle is responsible for 70%–80% of total insulin-stimulated glucose uptake, skeletal muscle IR is a major determinant of type 2 diabetes [5].

Interactions between genetic and environmental factors, overnutrition, and sedentary behavior promote the progression and pathogenesis of IR. In particular, the modifications that occurred in the global food system during the past 3-4 decades have created an “obesogenic” environment contributing to the increase of the obesity epidemic and consequent IR incidence increase. Unhealthy diet and physical inactivity are considered among leading causes of the same diseases characterized by IR. Currently, alleviating this condition is still one of the key strategies to treat [2, 6].

METF, a widely prescribed drug in type 2 diabetes, is being increasingly considered for treatment and prevention of sedentariness damages, as well as for the extension of healthy lifespan [7]. Recent data showed that long-term diet supplementation with METF extends healthy lifespan in* C. elegans* and in middle-aged male mice [8, 9]. In addition, our group demonstrated how acute METF treatment may induce the generation of neohypertrophic myotubes, by using an* in vitro *model of satellite cells (C2C12 cells line) [10].

Regular practice of physical exercise plays a very important role in maintaining a good state of health and physical well-being [11]. In particular, recent publications showed the active function of exercise in the reduction and counteraction of the mechanisms underlying muscle atrophy and degeneration related to the onset of peripheral IR [12, 13].

In exercising muscle, increased energy metabolism and ATP production is obtained by an increased glucose utilization. One of the most relevant metabolic effects of exercise is the enhancement of insulin action [14]. Many factors may contribute to increasing insulin sensitivity induced by exercise: a reduction in fat mass, an increase in muscle mass, and the increase of membrane-bond glucose transporters (GLUT4) in muscle cells [15].

The effects of physical exercise may have relevant implications in the prevention and treatment of metabolic diseases. In fact, by increasing insulin sensitivity, physical activity can reduce the risk of pathological conditions such as type 2 diabetes and metabolic syndrome [16].

Understanding the complex mechanisms that regulate insulin response and the onset of peripheral insulin resistance represents a primary goal in the treatment of diabetes and obesity complications, particularly by targeting skeletal muscle. Given the growing prevalence of the disease and the conditions of relative sarcopenia related to it, new therapeutic interventions are able not only to reduce the loss of skeletal muscle mass but also to stimulate muscle regeneration while preserving the physiology of viable muscle satellite cells become necessary.

In order to determine if Metformin could relieve the sedentariness damages, we studied METF effects in sedentary adult young mice, focusing our attention on METF ability to maintain mouse physical performance during submaximal incremental test.

## 2. Research Design and Methods 

### 2.1. Materials

Mouse C2C12 myoblasts were purchased from the European Collection of Animal Cell Cultures (ECACC). Reagents were purchased from Sigma Chemical Co. (Saint Louis, MO, USA). Primary antibodies against AKT (C-20), CAMKII (M-176), calnexin (H-70), ERK1 (K-23), ERK2 (C-14), GAPDH (FL-335), MyoD (C-20) Myogenin (D-10), MyHC (H-300), Myf5 (c-20), N-cadherin (H-63), p70S6 (C-18), SOD2 (FL-222), pERK1/2 (E-4-4) pp70S6 (sc-7984), peroxidase-conjugated secondary antibodies for Western blot analysis, and rhodamine-conjugated antibodies for immunofluorescence analysis were purchased from Santa Cruz Biotechnology (Santa Cruz, CA, USA). Primary antibodies phospho-AKT (Ser473) (D9E) XP and phospho-AMPK alpha (Thr172) (40H9) were purchased from Cell Signaling Technology (Danvers, MA, USA). Antibody against Phalloidin (Alexa Fluor 488 Phalloidin, molecular probes-Invitrogen).

### 2.2. Animal Studies

#### 2.2.1. Animals

Male C57BL/6 mice (*n* = 10), purchased from Charles River Laboratories (Boston, MA, USA), were used for the study at 12 weeks of age. All animals were kept on a 12 h/12 h light/dark cycle with unlimited access to standard rodent chow food and water. Mice were divided into two paired groups: one treated with METF and the other not treated (CONTR). Every week weight and blood glucose levels were determined. Blood glucose was measured in blood collected from the tip of the tail with a portable glucose measuring device (Bayer, Basel, Switzerland).

#### 2.2.2. METF Treatment

**Figure 1:**
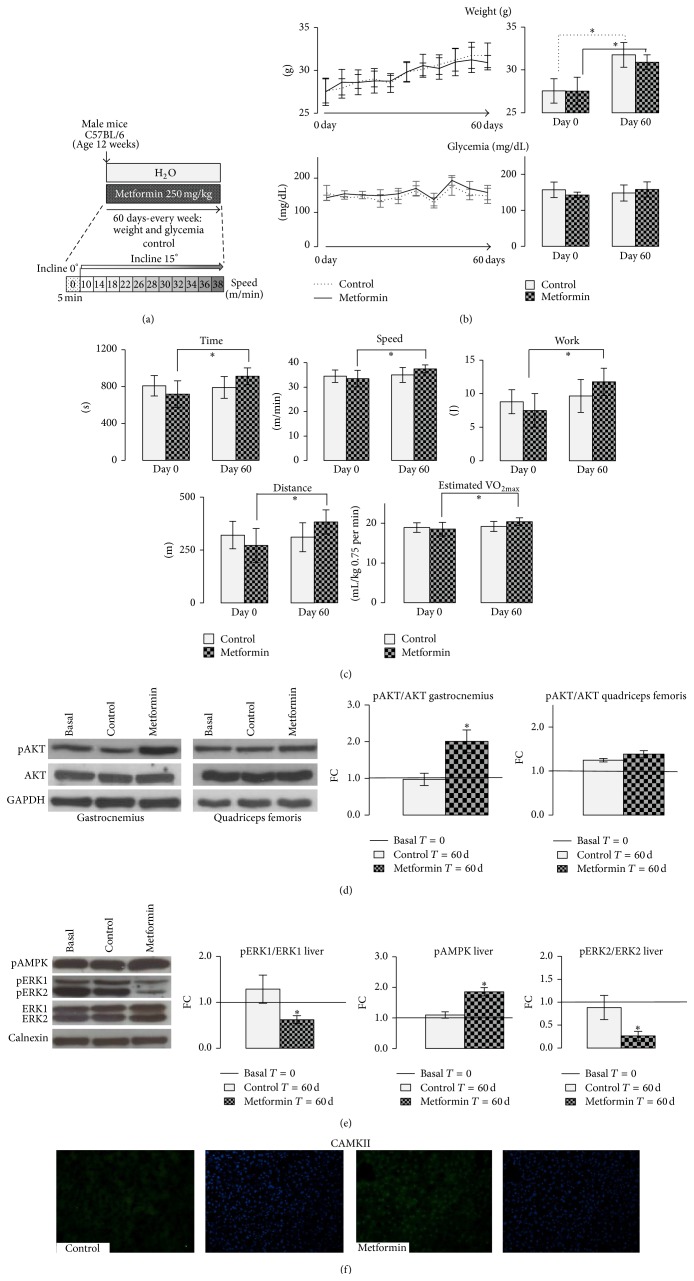
METF prevention of sedentariness induced damage in adult male mice C57BL/6. (a) Experimental protocol. (b) Weight and glycemia variation. (c) Muscular performance evaluation reveals that METF treatment ameliorated the muscular performance. (d) Muscle tissue analysis indicated that METF had a positive action on AKT activation. (e) Western blot liver analysis showed that METF increased AMPK activation and decreased ERKs activation. (f) Liver morphological studies: METF increased CAMKII signal. Representative immunoblots of analyzed proteins are shown. Scale bar 200 *μ*m. Significance: ^*∗*^ *p* ≤ 0.05.

METF (Sigma Chemical Co., Saint Louis, MO, USA) was added to the drinking water at dose of 250 mg/kg body weight per day, for 60 days. Control mice received water without METF. As reported in literature [17], our pilot study confirmed that C57BL/6 mice consumed 7 mL water per day; METF addition did not influence water consumption. Water and METF were changed daily and the dose adjusted to weight gain each week (Figures 1(a) and 1(b)).

#### 2.2.3. Muscular Performance Exercise Test

Muscular performance exercise was evaluated by a submaximal incremental test prior to and upon completion of the study. Briefly, mice were placed in adapted treadmill (Columbus Instruments) for 5 min at a 0° incline, and then treadmill speed was increased according to the scheme shown in Figure 1(a) with 1.5-min intervals at a 15° incline (Figure 1(a)). Animals run until exhaustion, which is defined as remaining on the shocker plate for more than 5 seconds. Food and water were unavailable to mice during any running sessions [18]. At the end of experiment, mice were sacrificed and tissues were harvested, frozen in liquid nitrogen, and stored at −80°C for further analyses.

This study was conducted in compliance with approved institutional animal care of the University of Milan.

### 2.3. Cell Culture Experimental Procedures

**Figure 2:**
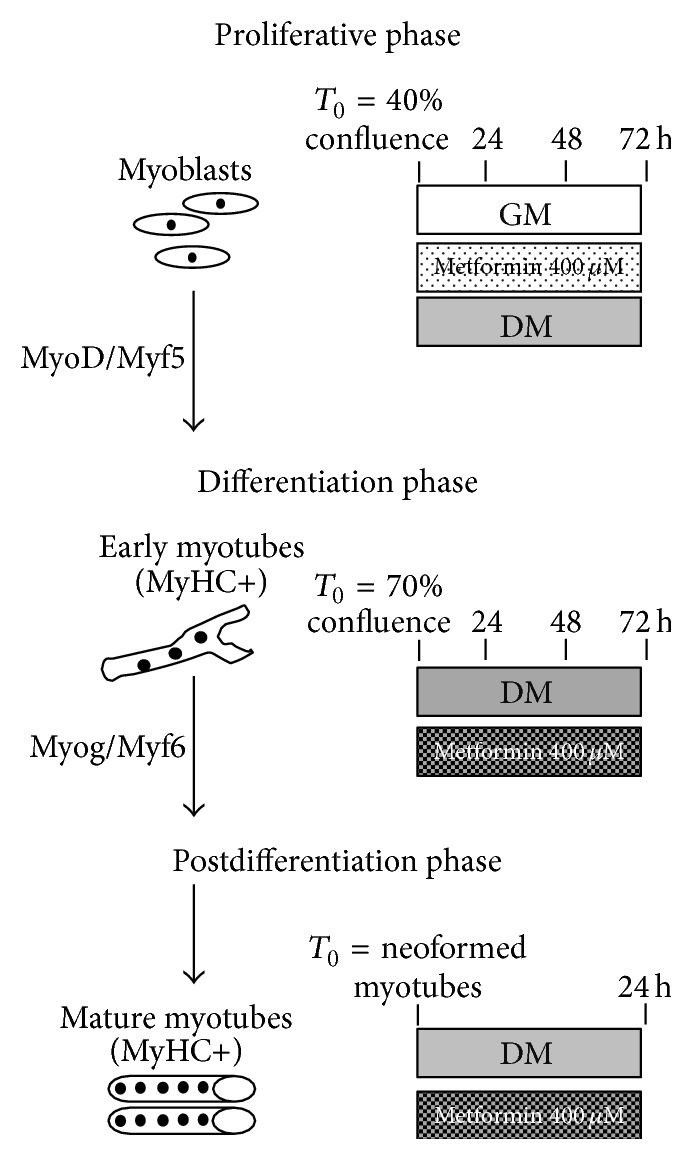
Experimental protocol* in vitro* studies. C2C12 cells in proliferative phase, in differentiation, and after differentiation were treated with 400 *μ*M METF.

C2C12 cells were maintained at 37°C in humidified 5% CO_2_ atmosphere in a growth medium (GM) containing DMEM (Dulbecco Modified Eagle Medium) supplemented with 20% (v/v) fetal bovine serum (FBS), 1% penicillin streptomycin, and 1% l-glutamine up to 70% confluence. Cell differentiation was initiated by placing 70% confluent cell cultures in differentiation medium (DM), containing DMEM supplemented with 1% horse serum (HS), antibiotics, and 1% l-glutamine. In our* in vitro* differentiation model, early myotubes appeared 24–48 hours (h) after serum starvation and neomyotubes formation was completed after 72 h [19]. Proliferating cells, myoblasts during differentiation process, and neomyotubes were treated with 400 *μ*m METF. In the control cells METF was not added to medium.  Figure 2 explains experimental study design in each phase of the protocol, with cell confluence percentage, treatments start time, and duration.

#### 2.3.1. Growth Curve and Cell Viability Test

To study METF action on C2C12 myoblast proliferation, we performed growth curve assay as described [20]. Briefly, C2C12 myoblasts were plated in 60 mm × 15 mm culture dishes at 40% confluence and grown in GM with or without METF and in DM. Medium was changed every 24 h and the experiment lasted until control cells achieved 70% of confluence (3 days). Every day, the cells were trypsinized, stained with trypan blue, and were counted using a hemocytometer and the average values for each single day were used to plot a growth curve. Cell viability was calculated by dividing the nonstained viable cell count by the total cell count. In addition, morphological changes were examined daily.

### 2.4. Western Blot Analysis

Protein extracts, performed as described elsewhere [21], were obtained from mouse tissues or cell cultures by using the following lysis buffer containing: 50 mM Tris/HCl, pH 7.4, 150 mM NaCl, 1% Triton X-100, 1 mM sodium orthovanadate (Na_3_VO_4_), 1 mM EDTA, 1 mM PMSF, 1 mg/mL aprotinin, 1 mg/mL leupeptin, and 1 mg/mL pepstatin.

Aliquots of 30 *μ*g supernatant proteins, quantified using Bradford method, were resolved on SDS-PAGE gel and transferred onto nitrocellulose membrane (Protran, Whatman Schleicher & Schuell). The membranes were incubated with specific primary antibodies and then with HRP conjugated anti-species-specific secondary antibodies. To confirm equal protein loading per sample, we used antibody anti-calnexin or anti-GADPH. Quantitative measurement of immunoreactive bands intensities, visualized by an enhanced chemiluminescence method (Amersham Pharmacia Biotech, Piscataway, NJ, USA), was performed by densitometric analysis using the Scion Image software (Scion Corporation, Frederick, MD, USA). Data were then converted into fold-changes (FC) of the controls [21].

### 2.5. Immunofluorescence Analysis

For tissues analysis, 7 *μ*m frozen cryosections was fixed in Formaldehyde 4% overnight at 4C. After that, slides were washed in PBS and incubated for 1 hour at room temperature with 10% horse serum in PBS with 0.05% Triton X-100 to block nonspecific binding sites, while C2C12 cells, fixed and permeabilized as described [22], were blocked with PBS containing 1% bovine serum albumin. Slides or cells were then immunostained with specific antibodies rhodamine-conjugated and nuclei revealed with DAPI staining. Slides were mounted with Moviol. Cells were observed using Nikon Eclipse 50I microscopy and images were captured using Nis-Elements D 4.00 software (Nikon Instruments Europe BV, Netherlands). Data were displayed and analyzed using Adobe Photoshop CS4. Live C2C12 cells were examined and images were acquired by phase contrast microscopy using the same microscope and digital system described above.

### 2.6. Statistical Analysis

All experiments were performed three times. Data are presented as the mean ± SD. Statistical significances were calculated using *t*-tests. Results were considered significant when *p* ≤ 0.05.

## 3. Results 

### 3.1. METF Prevention of Sedentariness Induced Damages in* In Vivo* METF Experiment

Previous data [8, 9] suggested the capacity of METF to extend lifespan in the nematode* C. elegans* and in middle-aged male C57BL/6 mice, improving healthspan mice.

We aimed to investigate the possible role of METF in the prevention of sedentariness induced damages. To achieve this scope, 12-week-old male C57BL/6 mice were chosen, maintained in a condition of total absence of exercise, and treated for 60 days with METF, added in water at the dose of 250 mg/kg body weight per day (Figure 1(a)).

We did not observe a significant modification in body weight between mice randomized to receive METF and those that did not receive METF (Figure 1(b)). A weight reduction was observed in both groups, comparing the beginning and the end of the experiment. In contrast, we did not observe significant effect in glycemia levels (Figure 1(b)).

METF effect on muscle performance evaluation revealed that METF treatment increased speed, time, work, and estimated maximal oxygen consumption (VO_2max_) of acute submaximal incremental exercise in the METF group as compared to their baseline values (Figure 1(c)). Overall, muscular performance capacity was therefore increased in the METF group with respect to the pretreatment condition, despite aging and despite the lack of previous exercise training performed whatsoever.

We evaluated METF effects on AKT. The AKT/mTOR pathway, crucial regulator of skeletal muscle mass, is upregulated during hypertrophy and downregulated during muscle atrophy [23, 24]. In gastrocnemius muscle of METF treated animals, AKT activation improved compared with controls (Figure 1(d)). In addition, a trend of an AKT activation was detected in quadriceps femoris muscle in METF treated mice (Figure 1(d)).

Next, we examined METF action on liver (Figure 1(e)). As already described in literature [25], METF increased AMPK activation in liver. Liver ERKs activation is associated with oxidative stress and metabolic dysfunctions, main features of obesity and diabetes [26]. Interestingly, ERKs activation was significantly decreased in liver of METF treated mice (Figure 1(e)).

Immunofluorescence analysis (Figure 1(f)) showed how METF positively modulates Ca^2+^/calmodulin dependent protein kinase (CAMKII) protein levels in liver tissue with respect to controls, suggesting an important role of METF in cellular calcium homeostasis.

All together, our* in vivo* data indicated that METF could ameliorate not only age induced damage but also sedentariness induced injury.

### 3.2. METF Positive Role in Proliferation, Differentiation, and Hypertrophy in Skeletal Muscle Myoblasts

We also tested METF action on skeletal muscle proliferation, differentiation, and hypertrophic process, using a C2C12 cell lines (Figure 2). C2C12 murine immortalized cell line provides a good* in vitro* model for the study of the major steps of myoblasts proliferation and differentiation [27, 28]. During C2C12 myogenic phenotype achievement, Myogenic Regulator Factors (MRFs) are expressed in a defined sequence; MyoD and Myf5 are primarily expressed, while Myogenin expression is only induced upon muscle differentiation [27, 28].

**Figure 3:**
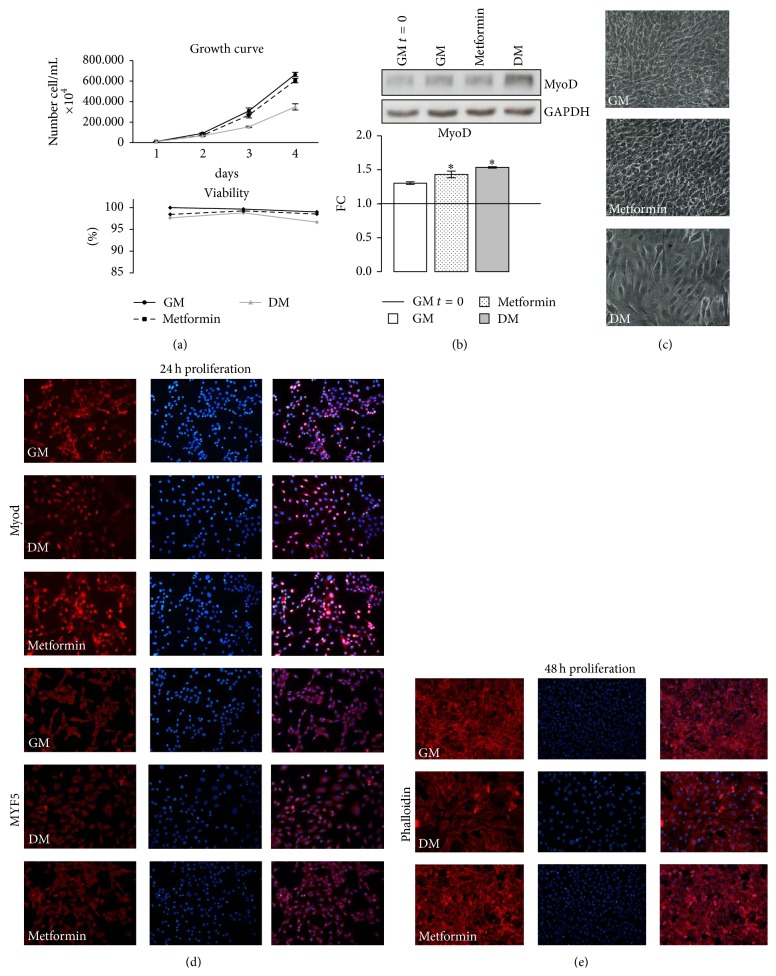
METF action on C2C12 proliferation phase. (a) METF did not modify C2C12 growth rate and did not induce cytotoxic effects. (b) METF increased MyoD protein content with respect to the control. (c) Phase contrast images at the end of proliferation phase. (d) Immunofluorescence analysis of early MRFs MyoD and Myf5 (24 h): METF enhanced myocytes committed. (e) Phalloidin assay at 48 h of proliferation. Representative immunoblots of analyzed proteins are shown. Scale bar 200 *μ*m. Significance: ^*∗*^ *p* ≤ 0.05.

400 *μ*M METF did not alter C2C12 proliferative potential and did not induce cytotoxic effects, as shown in Figure 3(a) and confirmed in phase contrast images in Figure 3(b). We also measured MyoD protein level. METF led to a significant rise in MyoD content, similarly to DM, compared with control (Figure 3(b)).

Immunofluorescence analysis during proliferation phase revealed that METF increased the protein expression of two key markers of early differentiation: Myf5 and MyoD, suggesting an important role in differentiation induction and promotion of the myoblast commitment to myotube (Figure 3(d)). To confirm this, Phalloidin assay (Figure 3(e)) showed that the cells lost their characteristic circular shape, typical of the active proliferation phase, to achieve a new elongated morphology.

**Figure 4:**
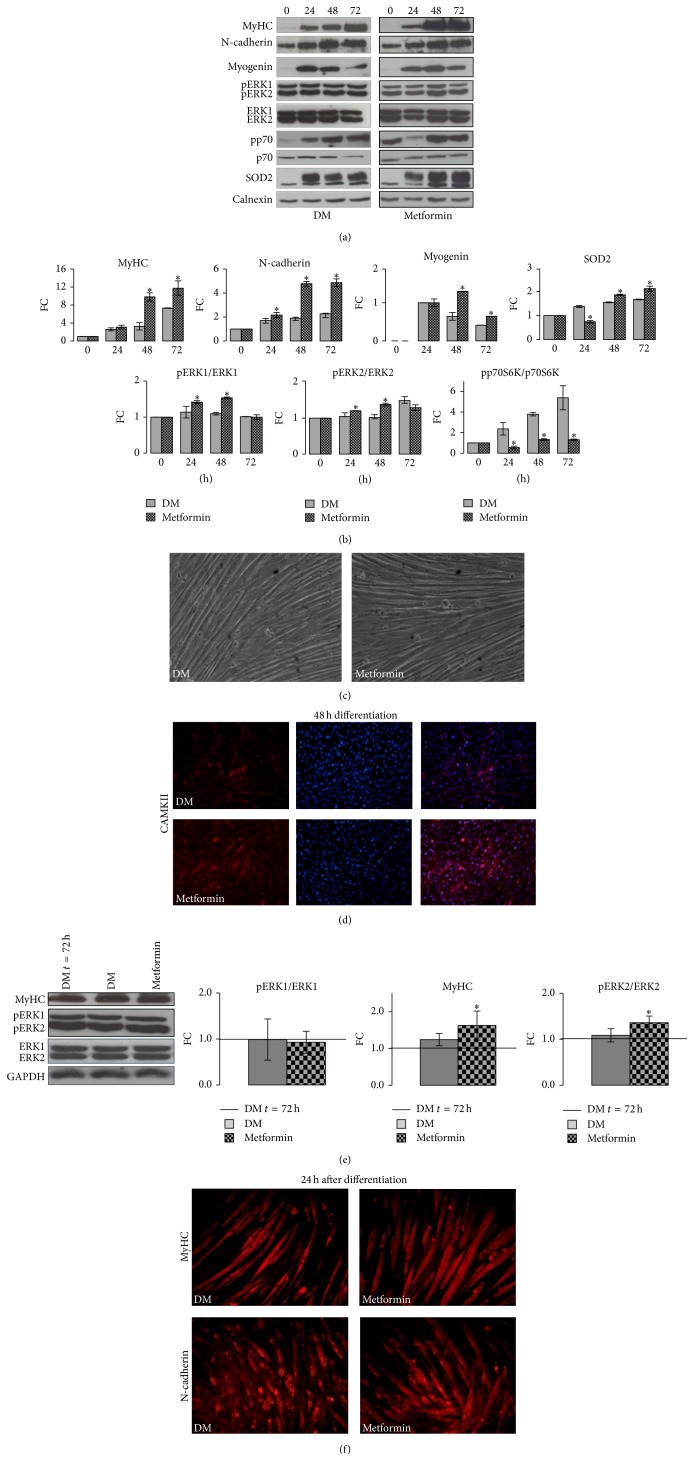
METF action on C2C12 differentiation and neoformed myotubes. (a) Representative Western blots for C2C12 cell lysates and the indicated antibodies. (b) METF enhanced myotube formation rising MyHC, Myogenin, and N-cadherin protein levels, improved mitochondrial antioxidant proprieties SOD2, activated ERK, and deactivated p70 signaling pathways. (c) Phase contrast images showed that differentiation efficacy is optimal in our model, since all competent myocytes became multinucleated myotubes. (d) At 48 h from differentiation induction, immunofluorescence analysis indicated positive role of METF in CAMKII protein. (e) METF action on neoformed myotubes: METF improved MyHC protein content and activated ERKs. (f) Immunofluorescence studies confirmed Western blot data of METF action in hypertrophic process. Scale bar 200 *μ*m. Significance: ^*∗*^ *p* ≤ 0.05.

We explored the METF effects on myotube formation. We analyzed METF action on differentiation (Figure 2), from the first phase of differentiation induction (24 h), middle phase (48 h), and to the end of the process (72 h), when all fusion-competent myocytes can form multinucleated myotubes [28]. As shown in Figure 4(b), METF treated cells were characterized by a significant rise in principal marker of myotube maturation, Myosin Heavy Chain (MyHC) protein levels with respect to control cells. The similar METF positive action was observed for N-cadherin, a central cytoskeletal protein involved in cytoskeletal rearrangement, required to the fusion of myoblast in new myotubes [29]. To corroborate our results, which indicated the active role of METF in differentiation progression, we analyzed Myogenin protein levels. METF enhanced Myogenin protein content and, in particular, its expression peak at 48 h (Figure 4(b)).

Protein level of Superoxide Dismutase (SOD2), an enzyme that efficiently converts superoxide to the less reactive hydrogen peroxide, was significantly increased in METF cells compared to control cells (Figure 4(b)). This result suggests that METF could counteract the damage caused by a sedentary lifestyle by strengthening the antioxidants mitochondrial functions.

Finally, we investigated METF action on the principal signaling cascades involved in skeletal muscle formation: ERKs and p70S6 kinase pathways [23]. METF enhanced differentiation process through ERKs activation, while it decreased p70S6 kinase pathway (Figure 4(b)).

After 48 h from differentiation induction, immunofluorescence analysis revealed that CAMKII protein expression was increased in neoformed myotubes treated with METF (Figure 4(d)).

Based on our previous data on effect of short time METF stimulation on the hypertrophic process [10], we studied the effect of long time METF treatment on neoformed myotubes (Figure 4(c)). 24 h of METF stimuli significantly increased MyHC protein levels (Figure 4(e)). As observed in all differentiation phases, METF effects were mediated by ERKs signaling pathways activation (Figure 4(e)).

Also in immunofluorescence images, we observed an important increase in MyHC and N-cadherin protein content after METF treatment on neoformed myotubes. Furthermore, METF treatment caused important morphological changes in terms of morphological parameters (myotubes length and diameter as shown in Figure 4(f)).

## 4. Discussion

The effects of physical exercise have relevant implications in the prevention and treatment of damage induced by “obesogenic environment” characterized by a positive balance between energy intake and energy expenditure. Moreover, physical activity represents a primary goal in the treatment of diabetes and obesity complications, in particular in skeletal muscle system. To corroborate the fundamental role of physical activity, the World Health Organization has identified physical inactivity as the fourth-leading risk factor for global mortality [30].

We investigated the potential effects of* in vivo* treatment with METF, currently candidate drug for lifespan extension, on the prevention of sedentariness induced damages. Specifically, we analyzed the METF capacity to improve mouse muscle physical performance.

The study of a protocol of exercise training in mice (Figure 1(c)) indicated that METF could have positive effects on muscular performance. Our results, obtained from studying a model of sedentary healthy mice, confirm the positive METF action on skeletal muscle function previously obtained in older mice models [8].

Several recent works suggest how this biguanide drug could be used in the prevention of aging induced damages [8, 9, 31]. In this perspective, we studied the key molecular regulators involved in common aging pathways [32]. AKT signaling is central in the regulation of muscle function and, in particular, AKT inactivation is associated with muscle damages induced by aging [23, 33]. We observed that METF increased the activation of AKT in gastrocnemius and quadriceps femoris muscles (Figure 1(d)), suggesting a hypothetical novel use of this drug not only in aging-related conditions, but also in sedentary-related damaged muscle conditions. Precisely, for the first time, our work showed how the beneficial effects of METF occur not only in groups already characterized by pathological conditions (e.g., obesity, diabetes, and aging-related disorders) but also in healthy sedentary populations.

An additional positive action was observed in METF treated mouse liver; METF deactivates ERK and promotes CAMKII signaling. ERK activation is crucial in favoring the development of several liver dysfunctions, such as liver fibrosis and hepatocellular cancer [34], while CAMKII pathways activation is fundamental to preserve liver functions [35]. From these data, it is reasonable to conclude that METF supplementation could be utilized to keep liver healthy. We demonstrated in healthy humans that constant aerobic physical exercise is the clue to avoid lipid steatosis [36].

To further clarify METF cellular mechanisms underlying the effects obtained in the mouse model, we studied METF action using an* in vitro* model of myoblasts, C2C12 cell line [27, 28]. This cell line represents the gold standard of immortalized cells to study not only myogenesis but also the hypertrophy process and its use has allowed us to investigate METF action on cellular pathways involved in muscle training, characteristic process of healthy subject.

First, we observed that METF did not modify C2C12 proliferation rate and viability (Figure 3(a)), confirming the possible use of this biguanide drug without side effects on skeletal muscle [37]. Those results have important implications since several tumors are associated with sarcopenia and cachexia [38], which might benefit from METF treatment.

METF accelerated myogenic phenotype acquisition and myotubes formation (Figures 3 and 4). After 24 h of METF stimuli on neomyotubes, we observed an increment in morphological parameters (Figure 4(f)), similar to our previous work where on acute METF treatment was administered [10]. This effect is mediated by ERK activation (Figure 4(e)). Our data indicate that METF should not be considered not only a drug capable of inactivating the cellular mechanisms related to muscle injury, but also a drug capable of activating cellular processes related to muscle strengthening and hypertrophy. We speculate that this METF capacity to enhance myotubes formation and hypertrophy could represent a possible explanation of data obtained,* in vivo*, in mice.

Finally, our results obtained during differentiation phases showed that SOD2 protein content is increased after METF stimuli (Figure 4(b)). The intracellular enzymes of the SOD family act as a primary line of defense to cope with the deleterious effects of ROS, thereby contributing to an overall decrease in oxidative damage. Lower SOD activity is associated with sedentary lifestyle, characterized by insulin resistance, suggesting that reduced capacities of antioxidant enzymes lead to increased oxidative stress in diabetes and obesity [39]. So, we hypothesize that METF could also act as an antioxidant agent. This potential effect needs further investigation.

## 5. Conclusions

In conclusion, our study reports several novel findings regarding the use of METF in a condition of absence of physical activity and specifically: (1) it improves mice physical aerobic performance; (2) it ameliorates myotubes formation, regulating the principal molecular mediators of skeletal muscle hypertrophy and atrophy; (3) it prevents oxidative stress damage, modulating ERK and SOD signaling.

The relevance of our results resides in a potential use of METF (or drugs with similar biological proprieties) to counteract the damages consequent to sedentariness either directly (acting on molecular targets involved in stress condition) or indirectly, by enhancing the known beneficial physical activity effects. In this framework, additional research is necessary, also in humans, to test combined therapeutical use of METF, exercise, and diet to prevent damages of sedentariness.
